# Spontaneous Spinal Epidural Haematoma Associated With Direct Oral Anticoagulants: A Descriptive Systematic Review

**DOI:** 10.7759/cureus.112438

**Published:** 2026-07-11

**Authors:** Shivam Ghosh, Adithya Shine, Manujan Sundaralingam

**Affiliations:** 1 Department of Surgery, Milton Keynes University Hospital NHS Foundation Trust, Milton Keynes, GBR

**Keywords:** apixaban, direct oral anticoagulants (doac), edoxaban, rivaroxaban, spontaneous spinal epidural hematoma (sseh)

## Abstract

Direct oral anticoagulant (DOAC)-associated spontaneous spinal epidural haematoma (SSEH) is an uncommon but potentially devastating haemorrhagic complication that can cause acute spinal cord compression. Current evidence is limited to case reports, case series, and accompanying narrative reviews, with no comprehensive systematic synthesis of the available evidence.

A systematic review was conducted in accordance with PRISMA 2020 guidelines. MEDLINE via PubMed was searched from database inception to March 2026 using predefined search terms, with additional studies identified through backward citation searching. Studies reporting atraumatic SSEH in adults receiving DOACs were included. Traumatic, iatrogenic, and non-epidural haemorrhages were excluded. Data extracted included patient demographics, DOAC type, clinical presentation, diagnostic pathway, management, and neurological outcomes. A descriptive synthesis was performed.

A total of 17 studies comprising 21 patients met the inclusion criteria. Rivaroxaban and apixaban were the most frequently implicated DOACs. Patients typically presented with acute neck or back pain followed by rapidly progressive neurological deficits, with several cases initially mimicking acute stroke. MRI was the most frequently utilised imaging modality, while CT also identified the haematoma in several patients, either alone or before subsequent MRI. Management was predominantly surgical decompression, although four patients were managed conservatively. Complete or partial neurological recovery was reported in 18/21 patients.

DOAC-associated SSEH is a rare but time-critical neurological emergency. Early recognition, prompt imaging, and timely intervention remain fundamental to optimising neurological outcomes. As the use of DOACs continues to increase, clinician awareness of this uncommon complication is essential to facilitate early diagnosis and appropriate management.

## Introduction and background

Spontaneous spinal epidural haematoma (SSEH) is an uncommon but important cause of acute neurological deterioration, characterised by bleeding into the epidural space with subsequent compression of the spinal cord. SSEH has an estimated incidence of approximately one in a million individuals per year [1] and may result in permanent neurological disability, particularly when diagnosis and intervention are delayed. Clinical presentation is often abrupt, with patients developing severe axial pain followed by rapidly evolving neurological deficits [2,3]. The extent of neurological recovery is strongly influenced by the severity of neurological deficits at presentation and the timeliness of intervention [4].

Direct oral anticoagulants (DOACs), including factor Xa and direct thrombin inhibitors, are widely used for the prevention and treatment of thromboembolic disease and have demonstrated favourable efficacy and safety profiles compared with vitamin K antagonists [5]. While intracranial and gastrointestinal haemorrhages are well-recognised complications of DOACs [5], spinal haemorrhages, including SSEH, remain rare and poorly characterised [6].

Current evidence regarding DOAC-associated SSEH is limited to isolated case reports and small case series. Although several reports have included narrative reviews of the existing literature, to our knowledge, no systematic review has comprehensively synthesised the available evidence regarding clinical presentation, diagnostic pathways, management strategies, and neurological outcomes in this population. Furthermore, delays in diagnosis and uncertainty surrounding anticoagulation management, including reversal strategies and the timing of anticoagulation resumption, continue to present important clinical challenges.

The present study therefore undertakes a systematic review of reported cases of atraumatic spinal epidural haematoma in patients receiving DOAC therapy, with the aim of describing patterns of clinical presentation, diagnostic pathways, management strategies, and neurological outcomes. By systematically synthesising the available evidence, this review aims to support earlier recognition and inform clinical decision-making in this time-sensitive and potentially reversible condition.

## Review

Methods

This descriptive systematic review was conducted in accordance with the Preferred Reporting Items for Systematic Reviews and Meta-Analyses (PRISMA) 2020 statement [7]. The review protocol was prospectively registered with PROSPERO (CRD420261298727).

Search Strategy

A systematic literature search was conducted in MEDLINE via PubMed from database inception to March 2026. The following search strategy was used: ("spontaneous spinal haematoma" OR "spontaneous spinal hematoma" OR "spontaneous spinal epidural haematoma" OR "spontaneous spinal epidural hematoma") AND ("apixaban" OR "rivaroxaban" OR "dabigatran" OR "edoxaban" OR "DOAC" OR "NOAC" OR "novel oral anticoagulants" OR "direct oral anticoagulant"). Both UK and US spellings (haematoma/hematoma, haemorrhage/hemorrhage) were included to maximise search sensitivity. No date restrictions were applied.

Backward citation searching (snowballing) of eligible studies was also performed to identify additional potentially eligible reports. Studies identified through this process underwent the same eligibility assessment as those identified through the primary search.

Eligibility Criteria

Inclusion criteria: Eligible studies included adults (≥18 years) presenting with an atraumatic SSEH or haemorrhage confirmed by MRI or CT imaging. Patients were required to be receiving a DOAC at or around the time of presentation; concomitant antiplatelet therapy was permitted and recorded where reported to reflect real-world prescribing practices. Only case reports and case series reporting primary patient data were included.

Exclusion criteria: Studies were excluded if they involved paediatric patients (<18 years), traumatic spinal epidural haematomas, or haematomas directly attributable to a spinal procedure or other iatrogenic cause (e.g. neuraxial instrumentation resulting in a haematoma at or adjacent to the procedural site). Non-epidural spinal haemorrhages, patients not receiving a DOAC (including those receiving warfarin, heparin or antiplatelet therapy alone), and non-primary literature (including systematic reviews, narrative reviews, editorials and commentaries) were also excluded.

Study Selection and Data Extraction

Titles and abstracts identified through the literature search were independently screened by three reviewers against the predefined eligibility criteria. Potentially eligible studies subsequently underwent full-text review to determine final inclusion. Studies identified through backward citation searching underwent the same screening and eligibility assessment process.

Data extraction was independently performed by three reviewers using a predefined framework. Extracted data included study characteristics, patient demographics, comorbidities, haematoma characteristics, diagnostic work-up, clinical presentation, management strategies, mortality, and neurological outcomes at discharge and at last follow-up, where reported. Neurological outcomes were categorised as complete recovery, partial recovery or no recovery according to the final neurological status reported by the study authors. Where follow-up data were unavailable, neurological status at discharge was recorded.

Data Synthesis

Due to the descriptive nature of the available evidence, which predominantly comprised case reports and case series, findings were synthesised narratively using descriptive summary tables. No quantitative synthesis or meta-analysis was performed.

Risk of Bias Assessment

The methodological quality of the included studies was independently assessed by three reviewers using the Joanna Briggs Institute (JBI) Critical Appraisal Checklist for Case Reports or the JBI Critical Appraisal Checklist for Case Series, as appropriate to study design [8,9]. Disagreements were resolved through discussion until consensus was reached. Overall methodological appraisal is presented, with domain-level assessments provided in Appendix 1.

Results

Study Selection

**Figure 1:**
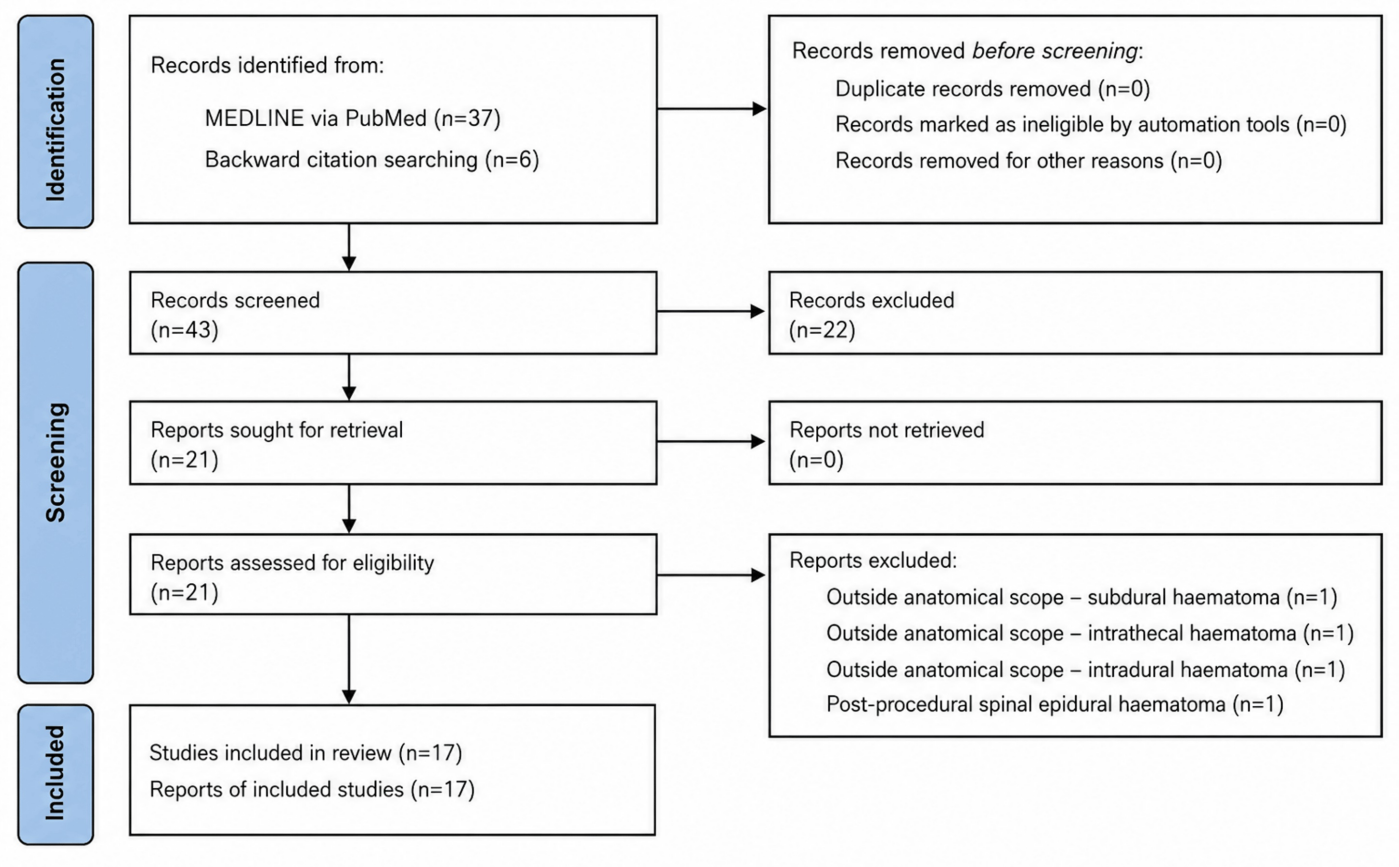
PRISMA 2020 flow diagram of study selection.

The systematic search identified 37 records through MEDLINE via PubMed with an additional six records identified through backward citation searching. No duplicate records were identified. Following title and abstract screening, 21 full-text articles were assessed for eligibility. Four studies were excluded following full-text review, comprising one subdural haematoma, one intrathecal haematoma, one intradural haematoma and one post-procedural spinal epidural haematoma. Seventeen studies met the inclusion criteria, comprising a total of 21 patients. The characteristics, clinical presentation, imaging findings, management strategies and outcomes of the included studies are summarised in Tables 1-2 [10-25]. A PRISMA flow diagram highlighting the study selection process is presented in Figure 1.

**Table 1:** Characteristics, presentation, imaging findings and neurological deficits of patients with DOAC-associated spontaneous spinal epidural haematoma. "Case No." denotes the case number assigned to each patient within a given study. For single-patient studies, this is recorded as 1. "DOAC ± AP", where AP denotes antiplatelet therapy. Recovery status was categorised according to the final reported neurological outcome. Where follow-up data were unavailable, discharge neurological status was used. Mortality was recorded as "No" where patients were reported to be alive at discharge or follow-up (NR = not reported). Neurological deficits represent the findings documented on neurological examination and may differ from the presenting symptoms because neurological deterioration frequently occurred between symptom onset and clinical assessment. DOAC: Direct oral anticoagulant; AP: Antiplatelet therapy; CT: Computed tomography; CTA: Computed tomography angiography; CTH: Computed tomography of the head

Study	Study Design	Case No.	Age	Sex	DOAC ± AP	Presentation	Neuro deficit	Imaging	Level
Mathais et al. (2018) [10]	Case Report	1	78	M	Dabigatran	Acute severe cervical and right shoulder pain	Right hemiparesis with pyramidal signs	CTH → CTA (identified) → MRI (characterise)	C4-T5
Goldfine et al. (2018) [11]	Case Report	1	74	M	Rivaroxaban	Gradual neck pain with progressive weakness and numbness below the clavicles and gait instability	Mild quadriparesis progressing to transient flaccid quadriplegia	CTA → MRI (identified)	Foramen magnum - C7
Ahmad et al. (2025) [12]	Case Report	1	75	M	Edoxaban	Lower head and neck pain, radiating to the shoulder with right arm and leg weakness	Right upper limb paresis and right lower limb plegia	CTH → CTA → MRI (identified)	C3-C5
Bamps et al. (2015) [13]	Case Report	1	70	M	Dabigatran	Acute severe cervical pain	Tetraplegia with sensory loss and autonomic instability	CT	C2-C4
Montalbetti et al. (2025) [14]	Case Series with literature review	1	71	F	Rivaroxaban	Progressive lower limb weakness and numbness	Complete paraplegia and anaesthesia	MRI	T10-11
		2	80	F	Apixaban	Acute neck pain with left-sided weakness	Left upper limb plegia and left lower limb paresis	CTH → CTA → MRI (identified)	C3-T1
		3	81	F	Dabigatran	Acute back pain with lower limb weakness and sensory deficit	Paraparesis, sensory deficit below T4, sphincter dysfunction	MRI	T3-T6
		4	79	M	Apixaban	Progressive right lower limb pain with weakness	Lower limb paraparesis with urinary urgency	MRI	L2-L3
Krupa et al. (2021) [15]	Case Report	1	84	F	Apixaban	Sudden severe back pain radiating to the left leg	Left lower limb weakness with sensory deficit below the knee	MRI	L1-L2
Ismail et al. (2017) [6]	Case Report with literature review	1	72	M	Rivaroxaban + Aspirin	Severe lower back pain with left-sided radiculopathy	Rapidly progressive paraparesis with saddle anaesthesia and urinary incontinence	MRI	T11-L2
Rahimizadeh et al. (2019) [16]	Case Report with literature review	1	66	F	Rivaroxaban + Aspirin	Acute neck and back pain	Brown-Séquard syndrome	MRI	C3-C6
Uddin et al. (2022) [17]	Case Report with literature review	1	86	M	Dabigatran + Aspirin + Clopidogrel	Acute onset weakness and gait instability	Quadriparesis with upper limb hyperaesthesia and ataxia	MRI	C2-T2
Jaeger et al. (2012) [18]	Case Report	1	61	F	Rivaroxaban	Severe thoracic back pain with bilateral lower limb weakness and numbness	Paraplegia with a T8 sensory level and sphincter dysfunction	MRI	C2-T8
El Alayli et al. (2020) [19]	Case Report with literature review	1	76	F	Apixaban	Acute back pain radiating to the left arm with progressive left-sided weakness and numbness	Severe left hemiparesis with sensory deficit	CT	C2-C7
Raeouf et al. (2020) [20]	Case Report	1	72	F	Rivaroxaban	Severe midline lower back pain	Acute paraplegia with sensory loss and sphincter dysfunction	CT → MRI (identified)	T10-L3
Mezzacappa et al. (2020) [21]	Case series	1	73	F	Apixaban	Sudden severe neck pain with progressive weakness and sensory changes	Right hemiparesis with right lower limb sensory deficit	CT (identified) → MRI (characterise)	C2-T3
		2	84	M	Apixaban	Sudden right neck and shoulder pain with progressive bilateral lower limb weakness	Severe quadriparesis with sensory loss below C6	CT (identified) → MRI (characterise)	C2-T1
Schoenmaekers et al. (2025) [22]	Case Report with literature review	1	61	M	Rivaroxaban + Clopidogrel	Acute cervicothoracic pain with bilateral hand paraesthesia	Paraparesis with bilateral lower limb sensory deficit and left upper limb hypoesthesia	CT (identified) → MRI (characterise)	CT: C5-T1, MRI: C2-T5
Theofanopoulos et al. (2021) [23]	Case Report with literature review	1	83	F	Apixaban	Sudden onset back pain with progressive lower limb weakness	Left lower limb plegia and right lower limb paresis with an L3 sensory level and urinary retention	CT (identified) → MRI (characterise)	T10-L3
Barkas et al. (2022) [24]	Case Report	1	72	M	Rivaroxaban	Sudden severe neck pain with progressive left-sided weakness	Left upper limb plegia, left lower limb paresis and urinary retention	CT (identified) → MRI (characterise)	C2-C4
Ozel et al. (2016) [25]	Case Report	1	69	F	Rivaroxaban	Cervical pain and weakness of the lower limbs and arms	Mild quadriparesis	CT	C2-C4

**Table 2:** Management, DOAC reversal, neurological outcomes and mortality of patients with DOAC-associated spontaneous spinal epidural haematoma. Time to surgery was recorded from symptom onset where reported. Surgery performed within 24 hours was classified as “Yes”. Where timing was unclear or not reported, this was recorded as NR (NR = Not reported). DOAC: Direct oral anticoagulant; FFP: Fresh frozen plasma; PCC: Prothrombin complex concentrate

Study	Management	Surgery within <24 h	Discharge outcome	Follow-up outcome	Recovery	Death
Mathais et al. (2018) [10]	C3-C7 Laminectomy - Idarucizumab pre-op	Yes	Complete neurological recovery	NR	Complete	No
Goldfine et al. (2018) [11]	Surgical evacuation of haematoma	Yes	NR	NR	NR	NR
Ahmad et al. (2025) [12]	Conservative (clinically stable with gradual improvement in motor strength and high risk for surgery)	N/A	Improved motor function	NR	Partial	No
Bamps et al. (2015) [13]	C2-C4 laminectomy - PCC pre-op	Yes	Complete recovery	NR	Complete	No
Montalbetti et al. (2025) [14]	Partial T9 and T11 and complete T10 laminectomy	No (48 h)	Partial sensory recovery	NR	Partial	No
	Left C6 hemilaminectomy	No (36 h)	Residual upper limb weakness	NR	Partial	No
	T3-T4 laminectomy	Yes	Complete recovery	NR	Complete	No
	L2-L3 hemilaminectomy	No (72 h)	Partial motor recovery of the lower limbs	NR	Partial	No
Krupa et al. (2021) [15]	T12-L2 laminectomy	No (48 h)	Improved motor function with mild residual weakness	Residual mild sensory deficit of the left lower limb	Partial	No
Ismail et al. (2017) [6]	T11-L2 decompressive laminectomy - FFP pre-op	Yes	Complete recovery	NR	Complete	No
Rahimizadeh et al. (2019) [16]	C3-C5 cervical laminectomy - FFP pre-op	No (36 h)	Partial neurological recovery	Complete recovery	Complete	No
Uddin et al. (2022) [17]	Conservative (Idarucizumab)	N/A	NR	Complete recovery	Complete	No
Jaeger et al. (2012) [18]	Conservative	N/A	Complete recovery	NR	Complete	No
El Alayli et al. (2020) [19]	C2-C7 laminectomy and spinal fusion	No (48 h)	Resolution of pain and minimal motor improvement of the left lower extremity	NR	Partial	No
Raeouf et al. (2020) [20]	Laminectomy (levels not stated) - FFP, Vit K and Factor VII pre-op	Yes	NR	No resolution of motor or sensory function in either of the lower limbs	No recovery	No
Mezzacappa et al. (2020) [21]	Partial laminectomy of C2 with C3-T2 laminectomies and right hemilaminectomy at T3 - PCC pre-op	Yes	NR	Residual weakness for right triceps extension	Partial	No
	C3-7 laminectomies with partial C2 and T1 laminectomies - PCC pre-op	Yes	NR	No improvement in neurological function	No recovery	No
Schoenmaekers et al. (2025) [22]	C6-C7 laminectomy - PCC pre-op	NR	No neurological deficits	Normal neurological status	Complete	No
Theofanopoulos et al. (2021) [23]	T10-L3 laminectomy	No	Mild residual distal left lower limb weakness	NR	Partial	No
Barkas et al. (2022) [24]	C2 and C3 hemi‐laminectomy, fenestration at C4 - PCC pre-op	Yes	Residual upper limb paresis	NR	Partial	No
Ozel et al. (2016) [25]	Conservative	N/A	Resolution of pain and motor deficits	Complete recovery	Complete	No

Methodological Quality Assessment

A total of 17 studies comprising 15 case reports and two case series underwent methodological quality assessment using the JBI critical appraisal checklists. Sixteen studies were assessed as being at low risk of bias, while one study was assessed as being at moderate risk of bias due to incomplete reporting of post-intervention clinical outcomes. A summary of the methodological appraisal is presented in Table 3, while detailed domain-by-domain assessments are available in Appendix 1.

**Table 3:** Methodological quality assessment of included studies using the JBI critical appraisal checklists.

Study	Overall JBI appraisal
Mathais et al. (2018) [10]	Low risk
Goldfine et al. (2018) [11]	Moderate risk
Ahmad et al. (2025) [12]	Low risk
Bamps et al. (2015) [13]	Low risk
Montalbetti et al. (2025) [14]	Low risk
Krupa et al. (2021) [15]	Low risk
Ismail et al. (2017) [6]	Low risk
Rahimizadeh et al. (2019) [16]	Low risk
Uddin et al. (2022) [17]	Low risk
Jaeger et al. (2012) [18]	Low risk
El Alayli et al. (2020) [19]	Low risk
Raeouf et al. (2020) [20]	Low risk
Mezzacappa et al. (2020) [21]	Low risk
Schoenmaekers et al. (2025) [22]	Low risk
Theofanopoulos et al. (2021) [23]	Low risk
Barkas et al. (2022) [24]	Low risk
Ozel et al. (2016) [25]	Low risk

Patient Characteristics

Among the 21 patients, the mean age was 74.6 years, with a slight female predominance (11/21, 52.4%). Rivaroxaban was the most frequently reported DOAC, accounting for nine cases (42.9%), followed by apixaban in seven cases (33.3%), dabigatran in four cases (19%), and edoxaban in one case (4.8%). Concomitant antiplatelet therapy was reported for four patients (19%), while the remaining patients solely received a DOAC.

Clinical Presentation

The presenting symptoms were varied but commonly involved neck or back pain accompanied by neurological deficits. Neurological manifestations were heterogeneous and included hemiparesis, paraparesis, quadriparesis, Brown-Séquard syndrome, sensory deficits, and bladder or bowel dysfunction. Individual presenting features and neurological deficits are outlined in Table 1.

Diagnostic Findings

MRI was the most frequently utilised imaging modality to confirm the haematoma. CT was also used, either as the initial imaging modality or in conjunction with MRI in several patients.

Anatomically, 14 of the 21 haematomas (67%) involved the cervical or cervicothoracic spine, each accounting for seven cases (33.3%). Thoracolumbar involvement was seen in three cases (14.3%), and isolated lumbar and thoracic involvement was observed in two cases each (9.5%).

Management

A total of 17 of the 21 patients (81%) were managed surgically, predominantly by laminectomy or hemilaminectomy, with nine patients (52.9%) undergoing surgery within 24 hours of symptom onset, while four patients (19%) were managed conservatively. Individual management strategies are summarised in Table 2.

Outcomes

Neurological outcomes were variable. Complete recovery was reported in nine patients (42.8%), partial recovery with residual deficits was also reported in nine patients (42.8%), and no neurological recovery was reported in two patients (9.5%). Outcome data were unavailable for one patient (4.8%). No deaths were reported among the patients for whom outcomes were documented.

Discussion

SSEH is a rare but potentially devastating neurological emergency that may result in permanent neurological disability, particularly if diagnosis and treatment are delayed. DOACs are increasingly used due to their favourable safety profiles in comparison with vitamin K antagonists [5], but reports of rare haemorrhagic complications such as SSEH have become increasingly recognised. Previous studies have emphasised the rarity of SSEH [1] and the importance of rapid diagnosis and intervention in achieving favourable neurological outcomes [26].

In this systematic review, 21 patients were identified from 17 studies with DOAC-induced SSEH. The majority of patients presented with acute neck or back pain accompanied by neurological deficits. Neurological deficits were heterogeneous, ranging from hemiparesis, paraparesis, quadriparesis, Brown-Séquard syndrome, sensory deficits, and sphincter dysfunction. Several patients presented with hemiparetic syndromes, making acute stroke the leading differential diagnosis. This finding is consistent with previous reports highlighting SSEH as an important stroke mimic [27,28]. The presence of acute neck or back pain with accompanying neurological deficits should prompt consideration of spinal imaging in anticoagulated patients presenting with possible stroke syndromes.

Among the imaging modalities, MRI was the most frequently utilised and remains the imaging modality of choice for the diagnosis of SSEH [1]. Several patients underwent an initial CT or CT angiography before MRI to evaluate for alternative diagnoses such as stroke or dissections. Although MRI remained the definitive modality, CT identified the haematoma or an epidural haemorrhagic lesion in some cases, highlighting its potential diagnostic value when MRI is not immediately available.

The majority of patients with progressive or significant neurological deficits underwent urgent surgical decompression with the aim of relieving spinal cord compression and minimising irreversible neurological injury. Previous studies have demonstrated that neurological outcome is influenced by both the severity of the presenting neurological deficit and the timing of surgical decompression, with earlier intervention generally associated with improved neurological recovery [26,29]. Although this review was not designed to evaluate prognostic factors, the predominance of surgical decompression among patients with significant neurological deficits is consistent with published recommendations favouring surgical intervention in neurologically impaired patients [1,29].

Despite the severity of presentation, neurological outcomes were largely favourable, with most patients experiencing complete or partial recovery, while only a small number had persistent severe deficits. These findings support previous observations that, despite severe neurological deterioration, there remains a possibility of meaningful neurological recovery, provided that the diagnosis and treatment occur before irreversible spinal cord injury develops [26,29]. However, despite intervention, several patients experienced incomplete recovery, highlighting the potential for long-term morbidity associated with severe spinal cord compression or delayed diagnosis.

Only a small number of patients were managed conservatively. Previous studies have suggested that favourable neurological outcomes may be achieved without surgery in carefully selected patients with mild, stable, or improving neurological deficits [30]. However, given the rarity of SSEH and the absence of comparative studies, the optimal selection criteria for conservative management remain unclear.

An additional challenge highlighted by this review is the lack of consensus regarding the management of DOAC therapy. Reversal strategies were limited, including prothrombin complex concentrates, fresh frozen plasma, and idarucizumab, and even the timing of DOAC resumption was inconsistently reported. Mezzacappa et al. [21] demonstrated contrasting approaches within the same case series, with apixaban being restarted on postoperative day 14 in one patient because of high thromboembolic risk, while the other patient had their apixaban permanently held because the risk of haemorrhage outweighed the benefit of stroke prevention. In most other reports, the timing of DOAC resumption was either not reported or highly individualised to thromboembolic risk, bleeding risk, and neurological status. As the use of DOACs continues to rise, further research is required to establish evidence-based recommendations regarding reversal and re-initiation of DOACs following SSEH.

This review has several limitations. All included studies were case reports or case series, resulting in a low overall level of evidence and an inherent risk of publication bias. Reporting of neurological examinations, imaging findings, management strategies, and outcomes was heterogeneous, limiting direct comparison between cases. Furthermore, given the rarity of DOAC-associated SSEH, no meaningful statistical analysis was possible, and the findings of this review should be interpreted as descriptive rather than definitive. The literature search was limited to MEDLINE via PubMed, supplemented by manual screening of reference lists (snowballing). Consequently, relevant studies indexed exclusively in other databases may not have been identified.

## Conclusions

This systematic review suggests that favourable neurological outcomes are achievable in many patients with DOAC-associated SSEH, although definitive conclusions regarding prognostic factors remain limited by the available evidence. MRI remains the diagnostic modality of choice, although CT may facilitate early diagnosis when MRI is not immediately available. Surgical decompression was the predominant treatment strategy in patients with significant neurological deficits, while conservative management was reserved for carefully selected cases. Given the limited evidence base and heterogeneity of the available literature, further studies are required to establish evidence-based recommendations regarding patient selection for conservative management, reversal strategies, and the optimal timing of DOAC resumption following SSEH.

## Appendices

Appendix 1

**Table 4:** Domain-by-domain JBI critical appraisal checklist assessment for included case reports. Q1 - Were the patient’s demographic characteristics clearly described? Q2 - Was the patient’s history clearly described and presented as a timeline? Q3 - Was the current clinical condition of the patient on presentation clearly described? Q4 - Were diagnostic tests or assessment methods and the results clearly described? Q5 - Was the intervention(s) or treatment procedure(s) clearly described? Q6 - Was the post-intervention clinical condition clearly described? Q7 - Were adverse events (harms) or unanticipated events identified and described? Q8 - Does the case report provide takeaway lessons? ✓ = Yes; ? = N/A; X = No

Study	Study design	Q1	Q2	Q3	Q4	Q5	Q6	Q7	Q8	Overall appraisal
Mathais et al. (2018) [10]	Case report	✓	✓	✓	✓	✓	✓	✓	✓	Low risk (8/8)
Goldfine et al. (2018) [11]	Case report	✓	✓	✓	✓	✓	✗	?	✓	Moderate risk (6/8)
Ahmad et al. (2025) [12]	Case report	✓	✓	✓	✓	✓	✓	✓	✓	Low risk (8/8)
Bamps et al. (2015) [13]	Case report	✓	✓	✓	✓	✓	✓	✓	✓	Low risk (8/8)
Krupa et al. (2021) [15]	Case report	✓	✓	✓	✓	✓	✓	✓	✓	Low risk (8/8)
Ismail et al. (2017) [6]	Case report with literature review	✓	✓	✓	✓	✓	✓	?	✓	Low risk (7/8)
Rahimizadeh et al. (2019) [16]	Case report with literature review	✓	✓	✓	✓	✓	✓	?	✓	Low risk (7/8)
Uddin et al. (2022) [17]	Case report with literature review	✓	✓	✓	✓	✓	✓	?	✓	Low risk (7/8)
Jaeger et al. (2012) [18]	Case report	✓	✓	✓	✓	✓	✓	?	✓	Low risk (7/8)
El Alayli et al. (2020) [19]	Case report with literature review	✓	✓	✓	✓	✓	✓	?	✓	Low risk (7/8)
Raeouf et al. (2020) [20]	Case report	✓	✓	✓	✓	✓	✓	✓	✓	Low risk (8/8)
Schoenmaekers et al. (2025) [22]	Case report with literature review	✓	✓	✓	✓	✓	✓	?	✓	Low risk (7/8)
Theofanopoulos et al. (2021) [23]	Case report with literature review	✓	✓	✓	✓	✓	✓	✓	✓	Low risk (8/8)
Barkas et al. (2022) [24]	Case report	✓	✓	✓	✓	✓	✓	?	✓	Low risk (7/8)
Ozel et al. (2016) [25]	Case report	✓	✓	✓	✓	✓	✓	?	✓	Low risk (7/8)

**Table 5:** Domain-by-domain JBI critical appraisal checklist assessment for included case series. Q1 - Were there clear criteria for inclusion in the case series? Q2 - Was the condition measured in a standard, reliable way for all participants included in the case series? Q3 - Were valid methods used for the identification of the condition for all participants included in the case series? Q4 - Did the case series have consecutive inclusion of participants? Q5 - Did the case series have complete inclusion of participants? Q6 - Was there clear reporting of the demographics of the participants in the study? Q7 - Was there clear reporting of clinical information of the participants? Q8 - Were the outcomes or follow-up results of cases clearly reported? Q9 - Was there clear reporting of the presenting site(s)/clinic(s) demographic information? Q10 - Was the statistical analysis appropriate? ✓ = Yes; ? = Unclear; X = No

Study	Study design	Q1	Q2	Q3	Q4	Q5	Q6	Q7	Q8	Q9	Q10	Overall appraisal
Montalbetti et al. (2025) [14]	Case series with a literature review	✓	✓	✓	?	?	✓	✓	✓	✓	✓	Low risk (8/10)
Mezzacappa et al. (2020) [21]	Case series	✓	✓	✓	?	?	✓	✓	✓	✓	✓	Low risk (8/10)
